# Capnocytophagia canimorsus – Severe sepsis in a previously well individual with no evidence of a cat or dog bite. A case report^☆^

**DOI:** 10.1016/j.amsu.2020.05.005

**Published:** 2020-05-14

**Authors:** Mohammad Umair Malik, Haleema Nadir

**Affiliations:** Burns and Plastics Department Wythenshawe Hospital, Southmoor Road, Wythenshawe, Manchester, M23 9LT, UK

**Keywords:** Case report, Capnocytophagia canimorsus, Sepsis, Burns and plastics

## Abstract

*Capnocytophagia canimorsus (C. canimorsus)* is a Gram-negative bacilli present in the gingival flora of canine and feline species. It is the second most common cause of infection following dog bites and contact with canine saliva, leading to severe sepsis in immunocompromised patients with no evidence of a breach to the skin. We present the case of a previously healthy 51-year-old male who presented with disseminated intravascular coagulopathy, acute renal failure with widespread haemorrhagic bullae and skin necrosis. He was treated empirically with broad-spectrum antibiotics for sepsis of unknown origin for several days before *C. canimorsus* infection was identified on blood cultures. Following this, a more detailed social history identified the vital historical detail that the patient owned 7 dogs. His purpura fulminans secondary to sepsis was managed conservatively with regular dressings by the Burns Department. Our experience demonstrates that *C. canimorsus* should be considered as a causative organism in patients presenting with sepsis of unknown origin after contact with domestic animals and treated with early antibiotic therapy.

## Highlights

• Consider canimorsus infection as a cause of sepsis in healthy patients with no evidence of dog/cat bites or scratches.
• Infection may be severe, causing DIC and multi-organ failure.
• The pathogen is slow-growing and difficult to isolate.
• Empirical treatment with broad-spectrum antibiotics is required alongside regular dressings to minimize transdermal fluid losses and maintain core body temperature.

## 1 Introduction

*Capnocytophagia canimorsus (C. canimorsus)* is a bacterial pathogen found in the gingival flora of canine and feline species. First identified in 1989, after a series of patients presented with a spectrum of fever, cellulitis, sepsis with multi-organ failure following dog bites or scratches. The blood cultures from these patients all identified a slow-growing Gram-negative bacillus, *C. canimorsus.* The majority of patients in this series were immunocompromised, with alcoholism or previous splenectomy the most common predisposing factors [1].

Here, we present a case of fulminant sepsis with multi-organ failure secondary to *C. canimorsus* in an immunocompetent patient with no evidence of cat or dog bite. This case report highlights the importance of thorough history taking in order to assess risk of underlying *C. canimorsus* infection, even in immunocompetent hosts.

This case report adheres to the 2018 SCARE guidelines [2].

## 2 Case report

A 51 year-old gentleman presented to our Emergency Department with sudden-onset central chest and abdominal pain. The patient had vomited six to seven times earlier that day and was suffering from intractable shaking of all four limbs. The patient stated that he had struggled to walk the short distance to the emergency department from his car. On examination, the patient appeared centrally cyanosed, and had a widespread purpuric rash with multiple blisters, haemorrhagic bullae and skin necrosis (Fig. 2). Although cardiovascularly stable on admission (systolic blood pressure of 116 mmHg, heart rate of 74 bpm), his respiratory rate was 18 breaths per minute and oxygen saturations were 75% on room air. His lactate was 4.3(normal range 0.5–1 mmol/L) and his core temperature on arrival was 33.1 °C (91.6 °F).

His past medical history was unremarkable, excluding a myocardial infarction in 2009, treated by coronary artery stenting. He had a 15-pack year smoking history and was known to have significant alcohol consumption. He lived at home with his wife and worked as a heavy goods vehicle driver, with part of his occupation involving handling of sewage waste.

**Fig. 1:**
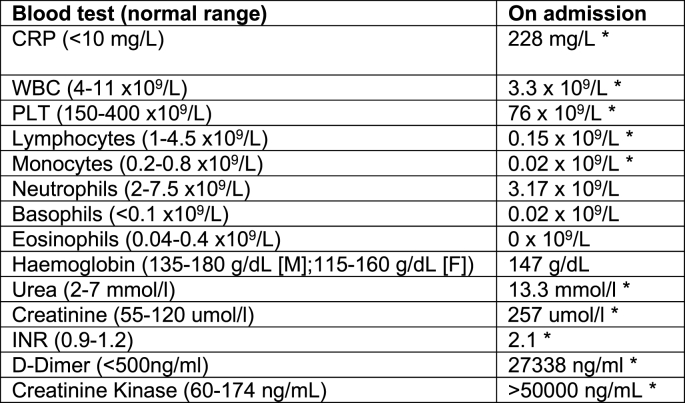
Blood tests taken on admission. Values outside normal parameters are highlighted (*).

Blood tests taken on admission (Fig. 1) demonstrated disseminated intravascular coagulopathy (DIC) secondary to sepsis. He was admitted to intensive care, intubated and placed on haemofiltration due to acute renal failure. Empirical broad-spectrum antibiotics (Tazocin and Clarithromycin) were commenced and these were subsequently modified to Meropenem, Teicoplanin, Clarithromycin and Metronidazole when initial blood cultures failed to culture a specific organism with a possible differential cause for his sepsis proposed as *leptospira*, contracted through his work with contaminated waste products. The patient remained intubated and ventilated for the following three weeks on ICU whilst his sepsis was managed. Skin biopsies demonstrated a non-specific skin reaction secondary to severe acute sepsis (purpura fulminans).

**Fig. 2:**
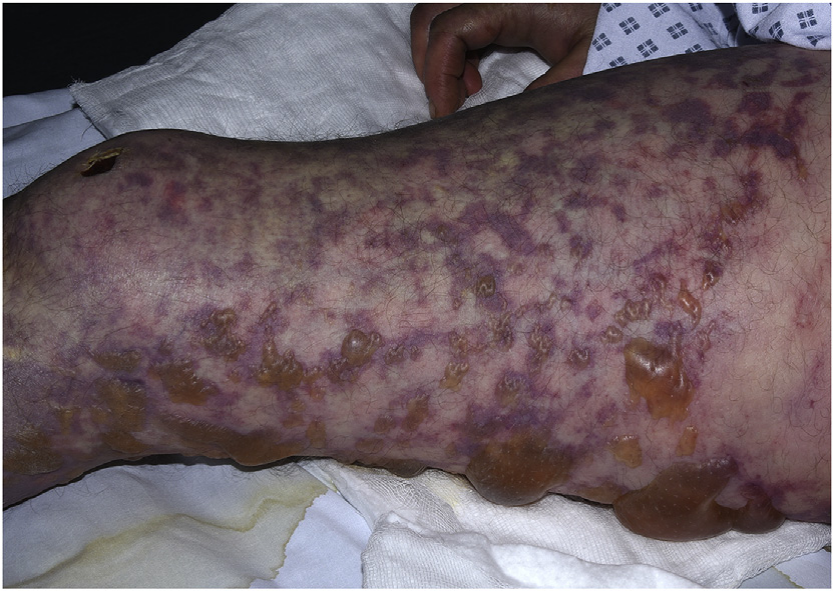
**Skin reaction.** Extensive purpuric rash with widespread blisters on the right leg.

**Fig. 3:**
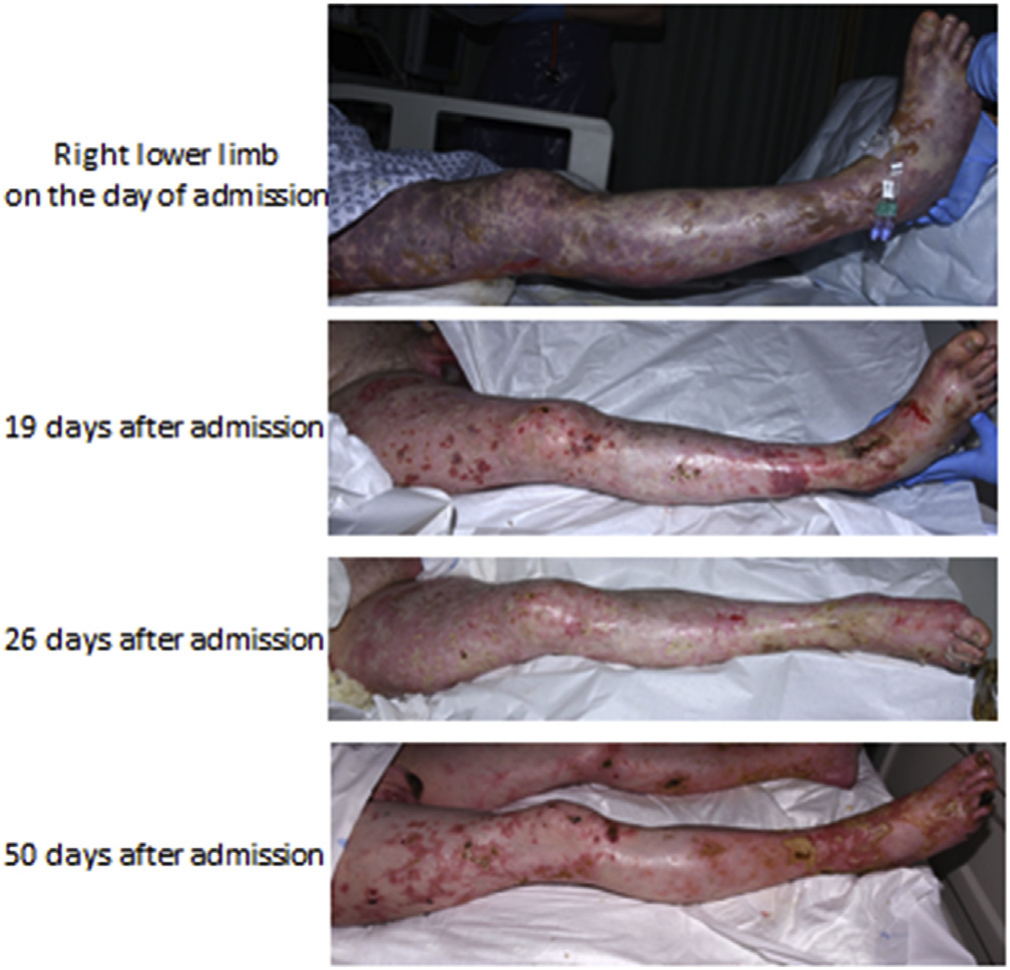
Gradual resolution of skin lesions across the period of admission.

At day 12 of admission, a slow growing Gram-negative bacillus was cultured from blood cultures. This finding together with the fact that the patient owned 7 dogs led to the identification of the causative organism as *C. canimorsus.* This diagnosis had not been previously considered since the patient had not been bitten by a dog nor had he been significantly pre-morbidly immunosuppressed. Treatment with intravenous Tazocin 4.5g was reinitiated three times daily for three weeks. Following treatment with Tazocin, the patient's haematological, renal and cardiorespiratory failure improved rapidly. His blistering rash and haemorrhagic bullae were managed conservatively with dressings by the Burns Department (Fig. 3). Further complications, including wound infections and pulmonary aspergilliosis resulted in total hospital admission of 7 weeks.

## 3 Discussion

*Capnocytophagia canimorsus* is a Gram-negative bacillus found in the saliva of 22–74% of healthy canine and feline species [1]. In total, 484 cases of disease due to this bacteria were reported, with a case mortality rate of about 26%, and 66% of cases in males [1]. Although 60% of cases occur after dog bites, 27% of cases may occur through more minor contact including licking, scratching or other close contact with cats or dogs [[3], [4], [5], [6]]. This particular point is important to consider, as while clinicians are concerned about open animal bites, we may fail to consider the potential of severe sepsis from transmission of animal saliva in other ways. Infection with the *C. canimorsus* causes variable symptoms, ranging from mild urticaria to fulminant sepsis, multi-organ failure or death [1,8]. 40% of *C. canimorsus* infection cases occur in healthy individuals. Individuals with previous splenectomy, immunosuppression or with significant alcohol intake have increased risk of infection as do smokers due to increasing plasma iron concentrations essential for bacterial growth [7,8].

*C. canimorsus* is difficult to isolate. It is a slow growing pathogen and can take up to 14 days to culture, explaining why in this case early blood cultures failed to isolate a specific bacterium [[9]^10^]. In this particular case, the sample was sent to a lab for PCR, which enabled the diagnosis to be made 12 days after admission. Capnocytophagia evades the immune response in a number of ways to produce a significant bacteraemia. It inhibits release of tumour necrosis factor-α and other proinflammatory cytokines produced by infected macrophages, as well as avoiding serum complement through its lipopolysaccharide structure. It has specific virulence factors, which enable it to resist phagocytosis and it can even exist intracellularly within leucocytes [[11], [12], [13], [14]]. These traits mediate its pathogenic behavior.

*C. canimorsus* infection is sensitive to a range of antibiotics, including beta–lactamase inhibitors, third-generation cephalosporins, carbapenems, doxycycline and clindamycin [7,15]. Our patient was treated with Tazocin intravenously for three weeks. The patients’ widespread haemorrhagic bullae, skin necrosis and blistering are together described as purpura fulminans, a non-specific reaction, caused by acute sepsis in this case. Upon questioning, the patient stated that he had suffered from purpuric rashes over his body for the last three years. The appearance of the rash coincided with when he first took ownership of his dogs.

The widespread nature of this skin condition required regular dressings to prevent insensible fluid losses, maintain core temperature and prevent superimposed bacterial infection.

## 4 Conclusion

The vast majority of published cases of *C. canimorsus* occur through animal bites. However, the combination of patient risk factors and frequent close contact with dogs, as in this case may also be sufficient to precipitate a fulminant Gram-negative sepsis. This case is also a rare example of a previously healthy patient requiring ICU admission secondary to *C. canimorsus* infection. This case illustrates the importance of a thorough social history particularly in cases of sepsis with unknown origin. If a patient presents with a similar history and blood cultures indicate the presence of a slow growing Gram-negative bacteria, *C. canimorsus* should be considered as a possible causative organism and treatment with a B-lactamase inhibitor should be started immediately in order to give the best chance of survival.

## Consent

Patient has consented to the use of images and data for publication.

## Ethical approval

Not required.

## Funding

None.

## Author contribution

Mohammad Malik Data collection, Writing of paper.

Haleema Nadir – Writing of paper.

## Registration of research studies

1. Name of the registry: Not Applicable
2. Unique Identifying number or registration ID:
3. Hyperlink to your specific registration (must be publicly accessible and will be checked):

## Guarantor

Mohammad Malik.

## Research registry UIN

Not applicable.

## Provenance and peer review

Not commissioned, externally peer reviewed.

## Declaration of competing interest

None.
